# Barriers and facilitators for physical fitness training in orthopedic geriatric rehabilitation. A qualitative study

**DOI:** 10.1080/09638288.2024.2314161

**Published:** 2024-02-15

**Authors:** Elizabeth M. Wattel, Franka J. M. Meiland, Johannes C. van der Wouden, Aafke J. de Groot, Cees M. P. M. Hertogh, Karin H. L. Gerrits

**Affiliations:** a Department of Medicine for Older People, Amsterdam UMC, location Vrije Universiteit Amsterdam, Amsterdam, Netherlands; b Amsterdam Public Health, Aging & Later Life, Amsterdam, Netherlands; c Department of Human Movement Sciences, VU University Amsterdam, Amsterdam, The Netherlands; d Merem Medische Revalidatie, Hilversum, The Netherlands

**Keywords:** Geriatric rehabilitation, physical fitness training, orthopedic, barriers, facilitators

## Abstract

**Purpose:**

The aim of this explorative, qualitative study is to identify factors that potentially influence the execution of physical fitness training in inpatient orthopedic geriatric rehabilitation (GR), from the perspectives of patients, their relatives and professionals.

**Materials and methods:**

In GR wards of skilled nursing facilities in the Netherlands, semi-structured interviews were held with triads of patients, their relatives and responsible nurses, and focus groups with members of the multidisciplinary teams. Verbatim reports were analyzed according to the framework method.

**Results:**

We found twelve categories of barriers and facilitators related to characteristics of the patients, their family, staff, training program and organization.

**Conclusions:**

The barriers and facilitators found largely correspond with those found for participation in exercise in related settings, but also show important differences. This overview of barriers and facilitators enables multidisciplinary teams to design improvements at the level of the organization and interventions, as well as at the level of the individual training program, tailoring it to the patient’s circumstances and needs. Further research should focus on weighing these barriers and facilitators to develop a feasible guidance for daily practice, as well as testing their effect on the adherence to existing physical fitness training guidelines.

## IMPLICATIONS FOR REHABILITATION

- Physical fitness training is important in reaching functional goals in orthopedic geriatric rehabilitation.
- Practicing outside of therapy can be stimulated by clarity about expectations, proper training equipment and tailored stimulation/assistance.
- Training volume and intensity should meet the guidelines, but be tailored to the patient.
- Characteristics of the program, staff and organization need to be considered as they impact the physical fitness training.

## Introduction

Geriatric rehabilitation (GR) is a multidimensional program of diagnostic and therapeutic interventions for older people with disabling impairments; for example, after hip fracture or elective orthopedic surgery [1]. The primary focus of GR is on restoring functional outcomes and social participation [1–3]. In practice, reduced physical fitness – such as aerobic capacity and muscle strength – is often a limiting factor in achieving functional outcomes in this aged target group [4,5]. Therefore, physical fitness should be an additional focus of the rehabilitation program. Despite the need for an increased focus on physical fitness training in GR, the implementation of evidence-based training guidelines is currently limited [6,7]. To facilitate such implementation, this study aims to understand the possible underlying factors that may influence the feasibility and effectiveness of physical fitness training in orthopedic GR programs.

General guidelines describe optimal training characteristics for improving physical fitness and recommend tailoring the training characteristics to the individual’s tolerance and preference [8,9]. In addition to these training characteristics, a well-tailored training program also considers factors that affect adherence to them. Therefore, it is essential to understand what factors are barriers or facilitators for physical fitness training in GR. In the Netherlands, inpatient GR wards are generally located in nursing homes, where also long-term care is provided, such as care for patients with dementia. There is a growing body of literature concerning factors that influence participation in physical activity and exercise in older adults in various settings, such as community dwelling, in-hospital and institutionalized older adults. Studies have found that adherence is stimulated by – for example – the patients’ willingness to exercise, support by others and the availability of exercise facilities [10–13]. Frequently mentioned barriers for exercise are limitations due to poor health status, heavy caregiver workloads and the layout of the (hospital) ward [10–13]. However, to date no research has focused on the specific facilitators and barriers for exercise adherence during inpatient GR.

Following a life event (fall or surgery), patients find themselves in a situation where they have to adapt to the consequences of e.g., the surgery and to a totally new setting where they reside during rehabilitation. For the majority of patients, participating in a structured rehabilitation program is a novel experience, so often they are not aware of what is expected from them concerning exercise programs, rehabilitation goals, improving their physical fitness and returning home. Therefore, the factors that influence exercise adherence might differ from those in the previously mentioned studies. Therefore, the factors that influence exercise adherence might differ from those in the previously mentioned studies. We expect to find specific factors for this setting, related for example to the fall and/or surgery, the return to their home, but also concerning the multidisciplinary collaboration.

In this explorative, qualitative study, we aim to identify factors that potentially influence the execution of physical fitness training in inpatient GR. Perspectives of patients, their relatives and GR professionals will be taken into account. To guide our exploration, we will take advantage of knowledge from implementation science, particularly the theoretical approach of determinant frameworks, that aim to understand and/or explain what influences implementation outcomes. Nilsen studied eight of the most commonly cited determinant frameworks and concluded that they are quite similar about the general types of determinants they account for. Therefore we used the general model of Nilsen, which distinguishes five types of determinants, such as characteristics of the intervention and characteristics of the user [14].

## Methods

### Design

A qualitative, explorative, descriptive design was used with semi-structured interviews and focus groups. The interviews were held with triads of patients, their informal caregivers and responsible nurses, and the focus groups with professionals from multidisciplinary orthopedic GR teams. This triangulation of methods and sources was chosen to create the most diverse and rich dataset possible, in a limited timeframe. Interviews allow for more detail about specific cases, particularly considering that for each patient three different involved persons were interviewed. Focus groups provide the benefit of interdisciplinary interaction where ideas arise and concerns are discussed. We reported according to the COREQ checklist [15].

### Setting and participants

This study is part of the Fit4Frail project, which investigates physical fitness training in orthopedic GR. The study was proceeded by a quantitative observational study of the current training of physical fitness in GR, which was performed in eleven GR wards recruited by the University Network of Organizations of care for older adults of Amsterdam UMC (UNO Amsterdam). In the current study, we aimed to include patients comparable to the participants in the observational study. Therefore we used the same in- and exclusion criteria as in the observational study. Patients aged 65 years or older, recovering from a trauma or elective surgery of the lower extremity and admitted for GR were included. Exclusion criteria included patients who 1) were non-Dutch-speaking; 2) gave no consent or were not mentally competent on this matter; 3) were unable to follow instructions; 4) were admitted for rehabilitation after lower extremity amputation 5) were not allowed to fully put weight on their legs; 6) had comorbidity that precluded from participation in the observational study; and/or 7) had an expected length of stay at the ward of less than two weeks. The latter three exclusion criteria were established to select patients that could perform all necessary measurement tests (like the six minutes walking test and the talk test) in the week of admittance and the week of discharge.

For this explorative study, we intended to include a convenient sample of participants, and recruited professionals and patients from three GR wards of the observational study, which were located in the middle and western parts of the Netherlands. For the semi-structured interviews, we aimed for six triads of patients, their informal caregivers and responsible nurses. For the focus groups, we targeted professionals from the multidisciplinary orthopedic GR teams, such as physicians, therapists and nurses, from each of the three wards. We aimed for three focus groups, one at each participating ward. This number of interviews and focus groups was considered feasible within the time frame of the project.

### Procedure

New patients were invited by their physician or therapist when they met the inclusion criteria. After obtaining the patient’s consent, as well as the consent of the informal caregivers and the responsible nurse, the interviewer contacted them to make an appointment for the interviews. Each participant was interviewed individually. For the focus groups, the GR professionals gave informed consent prior to the start.

### Ethical considerations

The study procedures were reviewed and approved by the Medical Ethics Review Committee of the VU University Medical Center (number 2019.345, Amsterdam, The Netherlands).

### Data collection

One semi-structured interview guide was composed for all participant groups, based on the implementation model of Nilsen, which summarizes the most commonly cited frameworks in implementation science, and distinguishes characteristics of the intervention, users and end users, the context and the implementation process [14]. As a behavior change (training) is needed to reach the GR goals in physical fitness training, the end user (the patient) characteristics of the model were extended with behavior-related items, such as self-efficacy and motivation. These items are derived from the integrated change model, which states that covert or overt behaviors are determined by a person’s motivation or intention to engage in a specific type of behavior [16]. The interview guide was tested in a pilot interview, and covered the topics of rehabilitation and exercising, persons involved in the rehabilitation and environmental factors. The interviewer was an elderly care physician who had recently followed a qualitative interview training, and had no professional or personal relations to the patients. Interviews were audio recorded.

For the focus groups, a topic list was based on the same model as the interview guide, covering the same topics. The focus groups were moderated by an experienced moderator and experienced elderly care physician (CH). The focus groups were audio recorded.

### Data analysis

Data were analyzed according to the framework method, a stepwise procedure for analyzing qualitative data [17]. All interviews and focus groups were transcribed verbatim. The researchers started with open coding, after which the codes were grouped into categories of barriers and facilitators fitting the characteristics of the Nilsen model. These categories formed a dynamic analytical framework. Quotes were considered to refer to physical fitness training if the terms “physical fitness training” or “training” were mentioned by the participants or if they mentioned activities that could influence endurance capacity or muscle strength [18]. For endurance capacity, this concerned activities that involved major muscle groups and were continuous and rhythmic in nature, like walking and cycling. For muscle strength, this concerned activities that involved certain repetitions or sets of force exertion against any resistance, like repeatedly lifting a leg or repeatedly standing up from a chair [18].

The interviews of the first triad (patient, informal caregiver, responsible nurse) and the first focus group were each coded independently by two researchers (DV en AM, in acknowledgements), after which any discrepancies were discussed until consensus was reached. All other transcripts were coded by one researcher each. New codes or adaptations to the codes were discussed. Categorization and the aggregation with the thematic model of Nilsen was undertaken in an iterative process. The codes were processed into Atlas.ti (version 22, Scientific Software Development GmbH, Berlin, Germany). For each category, the corresponding quotes were merged, summarized and classified as either barriers or facilitators for physical fitness training.

The resulting barriers and facilitators were presented to the members of the GR commission of the UNO Amsterdam in an online meeting. This commission comprises GR professionals of eighteen GR care organizations of the academic network (e.g., physicians, nurses and therapists). They were asked if the barriers and facilitators were recognizable and if other barriers and facilitators had been missed.

The level of experience of the professionals in the interviews and focus groups were rated according to their years of experience in GR: “junior” (0 through 5 years); “medior” (6 through 15 years) and “senior” (> 15 years). This information was adjusted to their quotes.

## Results

A total of fifteen interviews and two focus groups were conducted, including five patients, five informal caregivers and eighteen GR professionals. Due to the limited timeframe, a sixth patient could not be included. Due to the high workload in the GR wards, the third focus group was not planned. Interviews took place face to face at the GR ward between August and December 2019, and lasted between 14 and 56 min. The patients were all living alone, and had been admitted for fractures of hip or femur or (revision of) total hip arthroplasty. The characteristics of the participants are presented in Table 1. The focus groups took place at the GR ward in January 2020, and lasted one hour

**Table 1.** Characteristics of participants.

	Patients (*n* = 5)	Informal caregivers (*n* = 5)	GR professionals; nurses in interviews (*n* = 5)	GR professionals in focus groups (*n* = 13)
Median age (range)	86 (72-91)	53 (49-63)	48 (42-61)	47 (23-65)
Sex ratio (M:F)	0:5	1:4	0:5	0:13
Median length of stay at day of interview in days (range)	26 (21-29)			
Relation with patient Son Daughter		1 4		
Median professional experience in years (range)			29 (12-42)	18 (1-40)
Discipline Nurse/certified assistant nurse Physiotherapist Occupational therapist Exercise therapist Social worker Elderly care physician Quality advisor			5	3 3 2 1 1 2 1

*F = female; M = male; n = number*.

**Figure 1.**
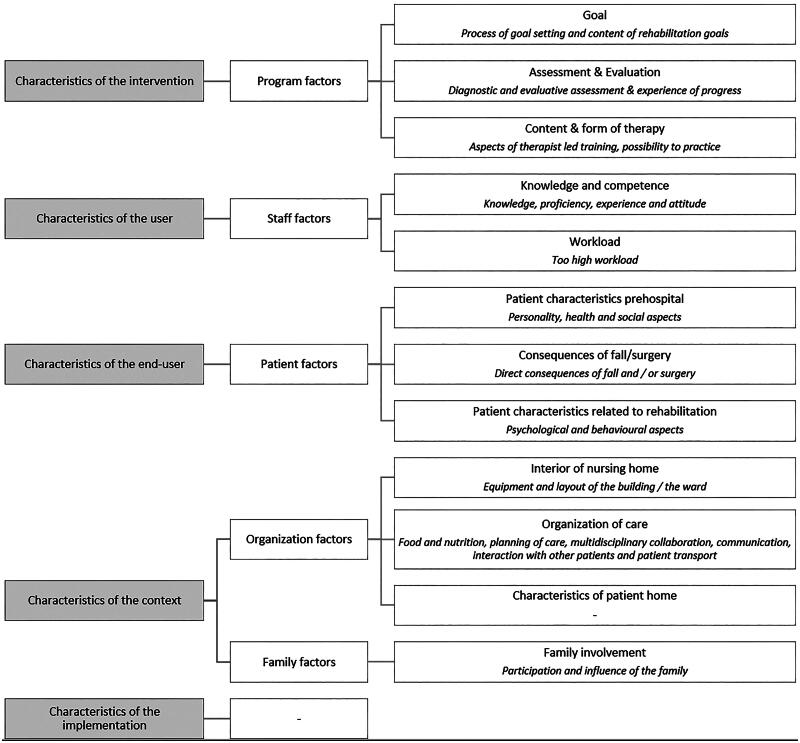
The Nilson model (left), the related factors (middle) and the categories of barriers and facilitators, including a brief description in italics (right).

Facilitators and barriers were found for four of the five characteristics of Nilsen’s model: the innovation (the program), the users (staff), the end users (patients) and the context (family and organization). Overall, twelve underlying categories of facilitators and barriers were identified. The members of the GR committee of the UNO Amsterdam recognized the results and believed that there were no missing categories. The model of Nilsen, the related factors and the categories of facilitators and barriers including their description are presented in Figure 1. In the next section, we further elaborate on the factors, barriers and facilitators that were expressed by the participants. An extensive overview of barriers and facilitators is presented in Table 2. As all informal caregivers were sons or daughters of the patients, from here onwards we will refer to them as “relatives,” while the GR professionals are referred to as “professionals.”

**Table 2.** Overview of barriers and facilitators for physical fitness training in orthopedic GR.

1. Program factors	
*Barriers*	*Facilitators*
1.1 Goal	
Lack of clarity about requirements to return home or perform a home visit. (REL)	Goals that account for premorbid functioning and home environment. (PRO, PT) Setting meaningful goals together with the patient, for both in- and outpatient rehabilitation. (PRO) Subdivision of goals into smaller sub-goals. (PRO)
1.2 Assessment and evaluation	
Insecurity about fracture healing. (PRO)	X-ray if there is uncertainty about fracture healing. (PRO) Rapid test to estimate level of functioning. (PRO) Using professional view/knowledge. (PRO) Using measurement instruments improves insight for both patient and GR professionals in: 1) prognosis of length of stay, 2) learning ability, 3) progression, 4) safety/fall risk, and 5) goal achievement. (PRO) Visible progress like the use of a normal chair instead of a wheel chair, or positive evaluation by therapist. (REL, PRO)
1.3 Content and form of therapy	
(Too) little physiotherapy (PT, PRO)/no weekend therapy. (PT) Limited space to exercise at home. (PRO) Limited practicing outside of therapy due to limited patient initiative, (PT, PRO) limited time of staff, (PT, PRO) restrictions for patient (not allowed to walk independently). (PT)	Optimal therapy frequency and intensity, e.g. High intensity and frequency for endurance training. (PRO) Strength training not on two subsequent days. (PRO) Good therapy content Home visit to formulate therapy goals. (PRO) For specific patient group (e.g., cognitively impaired) integration of training in ADLs. (PRO) Practicing outside of therapy Role of nursing staff: assisting/stimulating/slowing down, removing wheelchair/commode chair. (PRO) Equipment on ward such as a home trainer. (REL) Positive effect on self-efficacy. (PRO)
2. Staff factors	
*Barriers*	*Facilitators*
2.1 Knowledge and competence	
Lack of professional attitude (e.g., in case of lacking chemistry between patient and GR professional/proper treatment of patient. (REL, PRO) No insight into patient’s progression. (PRO)	Experienced GR professionals. (PRO) Proficiency in: Motivating/inviting and giving confidence: being able to adapt the treatment to the patient. (REL, PRO) Accounting for patient diversity, and adjusting frequency and content of therapy to patient’s needs. (PRO) Knowledge of: How to assess the appropriate training level. (PRO) Interpreting pain in a patient: is something wrong or can we continue exercising? (PRO)
2.2 Workload	
Lack of time/(experienced) staff to help patient practice outside of therapy. (PT, PRO) Patients don’t ask for help or to practice when they see that staff are busy. (PRO)	Patient tries to be self-supporting when there are not enough staff members. (PT)
Patient factors	
*Barriers*	*Facilitators*
3.1 Pre-hospital patient characteristics	
“Loneliness.” (PRO) Comorbidity that influences rehabilitation (e.g., cognitive impairment, pulmonary or cardiac problems (COPD, heart failure). (PT, PRO) Character and temperament, e.g., externalization of locus of control, too polite to ask for help, too easy-going. (PT, PRO) Cultural diversity, e.g., more worries about pain in non-Western background. (PRO)	Good initial fitness (at home). (PT, PRO) Character, e.g., initiative, flexibility. (PT, PRO) Independence. (PRO)
3.2 Consequences of fall/surgery	
Pain and doubts whether pain is alarm signal. (PT, PRO) Problems in in fixation of screws/position of the leg. (PT, PRO) Restrictions to bear full weight on affected leg, including restrictions to perform strength training. (PRO)	
3.3 Patient characteristics related to rehabilitation period	
Anxiety, e.g., about falling (again). (PRO) Insecurity, e.g., due to pain, or if a skill is not practiced enough. (PT, REL, PRO) Inactivity, e.g., not asking to practice, sitting all day in one’s room. (PRO)	Self-confidence. (PT, REL) Motivation, incentive. (PT, REL, PRO) Adequate expectations and preparation concerning length of stay, level of functioning at discharge, do’s and don’ts (generally better in elective patients). (PT, PRO) Role of patient: desire and capability to keep control/responsibility, e.g., practicing outside of therapy, clarifying what is important and what one’s own goals are. (P, PRO) Social interaction with others. (PRO)
4. Organization factors	
*Barriers*	*Facilitators*
4.1 Interior of nursing home	
Twin rooms: little space to move around. (PRO) Location of nurse’s office: no insight into patients practicing outside of therapy. (PRO) Too long corridors and walking distances, encouraging to take wheelchair. (REL, PRO)	Private bathroom stimulates to do ADL oneself, and thereby walking short distances. (PRO) Shared bathrooms: if occupied then walk to the next (PRO) Rooms that are inviting to go to and to practice/exercise. (PRO) Home trainer, leg press, hand rail in the corridors etc. in GR ward (PRO) Preconditions: spacious corridors (REL, PRO)
4.2 Organization of care	
Planning of care Many not-patient-related tasks and activities, e.g., new patient files, meetings, education. (PRO) Multidisciplinary collaboration Lacking or contradictory transfer documents from hospital. (PRO) Multidisciplinary meeting without patient. (PRO) Communication between various stakeholders Lacking communication with family about opportunities for family to participate in exercise. (REL) Transport to group therapy meetings after discharge. (PRO)	Food and nutrition Tasty and nutritious (enough protein) food. (REL, PRO) Planning of care Planning for assistance practicing outside of therapy (“walking list”). (PRO) Incorporate practicing in ADL (e.g., putting wheelchair further away). (PRO) Multidisciplinary collaboration Multidisciplinary team (where everyone is involved, not only physiotherapy, occupational therapy, doctor, nurses, dietician, speech therapist). (PRO) Team meetings to align approach, therapy intensity, practicing outside of therapy. (PRO) Communication between various stakeholders Communication with patient and family about: Functioning, progress, need for longer stay, possibility for family to join therapy sessions. (REL, PRO) Clarity about what to expect in GR for patients and family, e.g., clear agreements, a plan towards discharge. (PRO) Interaction with other patients: seeing other patients exercising. (PRO)
4.3 Characteristics of patient’s home	
Poor accessibility (many stairs) discourages practicing outdoors. (PRO)	
5. Family factors	
*Barriers*	*Facilitators*
5.1 Family involvement	
Don’t know that they are allowed to help. (REL) Put on the brakes. (PRO) Fear of overexertion in case of pain. (PRO) More nurturing than needed. (PRO)	Family practicing with patient. (REL, PRO) Collaboration between family and GR professionals: early involvement, providing information. (PRO) Stimulus for patient. (REL)

The stakeholder group that mentioned barriers/facilitators is mentioned in parentheses: PT = patient, REL = relative, PRO = GR professional.

### Program factors

#### Goal

A relative stated that the absence of a proper goal hindered adequate training. *[Relative 03]: “It seems to run sort of improptu, like ‘oh, she has to do this today, so let’s just do a quick climb up those stairs.”* Professionals stated that goals are important to motivate patients for training. *[Moderator]:” For proper training, how important is it to set goals?” [Occupational therapist FG2; junior]:” It covers motivation. Yes, that also tells the client what to work towards.”*

#### Assessment and evaluation

Professionals mentioned assessment and evaluation as factors influencing physical fitness training, such as the use of quick tests and formal tests to set and monitor fitness goals, as well as the use of visible progress that motivates the patient. *[Occupational therapist FG1; senior]” It sometimes may add encouragement for them to think ‘this is where I got yesterday, I really want to be able to walk a bit further.’”*

#### Content and form of therapy

All participant groups considered the possibility of practicing outside of therapy influencing physical fitness training. Interestingly this possibility was influenced by other types of factors, such as patient and staff factors, which shows an interrelatedness between factors that can occur. For instance, patients mentioned (a lack of) own initiative and – if they needed help – limited time of staff. *[Patient 01]: “And perhaps I should also have asked, like, ‘guys, do you have time to walk?’ Perhaps I’m slow in such things.” [Patient 05] “And they just say ‘busy, busy, busy, busy’.” Another barrier, mentioned by* professionals is that patients often do not see that participating in daily tasks is also part of the rehabilitation program and can help to improve their fitness and functioning. Professionals state that ‘everything is rehabilitation’. *[Occupational therapist FG1; senior]: "I say, ‘yes you’re talking about 30 min of therapies 2, 3 times a week, but this is also therapy, getting out of bed by yourself, standing by yourself, turning by yourself, walking.’ And… those… those are things that you may have to try to instil in people at times."*

Some quotes concerned therapist-led training. According to patients, good training equipment encourages fitness training, while the absence of therapy at the weekend hinders training. Professionals discussed the optimal frequency of muscle strength training and stated that it should not be undertaken on consecutive days.

### Staff factors

#### Knowledge and competence

Professionals mentioned the proficiency in motivating and inviting for physical fitness training. They also mentioned the knowledge of how to assess the appropriate training level and the interpretation of pain in a patient, which they related to the experience of professionals. Relatives experienced that the lacking professional attitude of a staff member hindered physical fitness training. They viewed proficiency in tailoring the training program to the patient’s specific needs as a crucial enabling factor. *[Relative 05]: “So, look at each person’s traits and go from there. Not treating everyone the same… no… just ‘your need this, you need that.’”*

#### Workload

This factor concerned the lack of time of staff hindering physical fitness training, which was expressed by both patients and professionals.

### Patient factors

#### Pre-hospital characteristics

The personality of the patient – named by patients and professionals – was mentioned as an influencing factor and was further specified; for example, such as being too easy-going*. [Responsible nurse 05; senior]:*” *I say, ‘put your chair on the brake’ and say ‘you want to go for a nice walk’. But some people go for it, but some people think: ‘well, nice try, but I’ll let myself be driven in my chair, thank you very much.’”*

Both physical and cognitive comorbidity are seen as barriers for physical fitness training. *[Patient 03]: "Look, they […] then found that I had a slightly murmuring heart, so maybe that’s why I also get short of breath faster with that rowing exercise." [Occupational therapist FG2; junior]: "And if someone is cognitively just fine, then they often also go and do extra homework and exercises and they also understand the instructions they were given, so then they can also rehabilitate by themselves, [better] than if someone is hampered in that respect."*

Another factor named by professionals is general fitness prior to hospitalization. *[Occupational therapist FG1; senior]: “Look, sometimes someone still cycled 10 kilometres each day, […] they can take on a higher load than an old girl who more or less only walked to the front door to empty the letterbox and back again.”*

Social aspects that professionals considered to influence physical fitness training include cultural differences in coping with pain, financial barriers for utilizing professional training after discharge home, and finally loneliness and its associated lacking incentive for training.

#### Consequences of fall/surgery

Professionals considered weight-bearing restrictions after surgery a barrier for muscle strength training. *[Physiotherapist FG2; senior]: "The use of strength training equipment is actually a contraindication for people with orthopedic rehabilitation, the first few weeks."* Professionals also mention the fear of falling as a barrier for physical fitness training. This can be directly associated with the fall but also have other causes, and is therefore also mentioned as a patient characteristic related to rehabilitation. Pain and loss of fitness after surgery are both mentioned as barriers for training by patients and professionals. *[Patient 03]: “I try, but I get short of breath, so it really does take me quite some effort.”*

#### Patient characteristics related to rehabilitation

Professionals mention fear (of falling) as a barrier for physical fitness training, where fear of falling also fits under consequences of fall/surgery. Motivation is a facilitator mentioned by all participant groups, which can relate to motivation towards a specific rehabilitation goal or the more general motivation to recover. *[Relative 03]: "So, she tells herself, in her head, ‘[…] in a few months I’m going to do that again’, well, I think that’s going to help with rehabilitation.” [Patient 03] “Then they say there’s someone at the back, that you can always call if you can’t manage. But then I won’t give in, […] I’ll then keep working on that rowing machine."* Moreover, besides those more psychological characteristics, inactivity is seen as a behavioral barrier for physical fitness training, such as patients sitting all day in their room during their rehabilitation or after discharge at home, when the incentive for rehabilitation declines.

### Organization factors

#### Interior of nursing home

Patients, relatives and professionals mentioned spaces that were inviting to exercise as facilitators for physical fitness training, such as spacious corridors, or a restaurant where lunch and dinner are served. However, if the walking distances are too long, they become a hindering factor for physical fitness training. *[Relative 03] "My mother was at the very end of the corridor and […] when they say, ‘well, come down to eat’ and you don’t have that much strength yet, you don’t have a spot or a chair or something similar anywhere where you can take a breather."* Professionals mentioned the presence of equipment – like a home trainer or a hand rail in the corridors – as enablers for physical fitness training.

#### Organization of care

One of the topics in this category is that tasteful and nutritious food facilitates physical fitness training. *[Relative 02]:” She is also eating good regular protein again here and everyone says that is good for her, for her muscle building."* Both relatives and professionals highlight the importance of good communication; for example, about the possibility for the family to participate in exercise. *[Relative 03]: “Perhaps we can mean something in that respect, that maybe we can go and walk outside for an extra hour, so to speak. And, in fact, I now have absolutely no idea whether that is expected of us at all."* Other subtopics are only mentioned by professionals; for example, structural planning of walking with patients on the ward, such as at a fixed time of the day, or incorporating training in daily activities. Furthermore, professionals mention the importance of multidisciplinary collaboration. *[Occupational therapist FG2; junior]: “… Can the nursing staff mobilize a little more with the walker? When do we actually remove the wheelchair completely, that someone must mobilize freely. That’s how you coordinate with each other, but also with the client*.”

#### Characteristics of patient home

This topic is named by professionals, and concerns how therapy goals are determined by characteristics of the patient home, such as the need to climb stairs, as well as how these characteristics influence physical fitness training at home. *[Assisting Nurse FG1; senior]: "And that’s of course here in Y, if you live on the 4th floor, […] then you’re just happy to be back in your chair on the 4th floor and then you think, like, ‘yes, the kids will do the shopping.’"*

### Family factors

#### Family involvement

Relatives believe that they can support physical fitness training by stimulating a patient to exercise or by practicing with the patient themselves. *[Relative 04]. "That’s what I tell her too, like, ‘even if it’s only for 10 min, go for a bike ride, that will help your muscles in your thigh.’”* On the other hand, not knowing that you are allowed to help is seen as a barrier by others. *[Relative 01] “Yes, I don’t know of that possibility at all. […] no one says like, ‘gosh, hey, if you want to, you can go and practice with madam in the room.”* This aligns with professionals, who see a positive collaboration with the family as a facilitator for physical fitness training. By contrast, family can hinder physical fitness training by being too cautious, and by putting on the brakes; for example, due to the fear of a premature discharge. *[Physician FG2; senior]* “*They very often put on the brakes a little, because they are incredibly afraid that dad, mum will be sent home too early […] so, to be frank, I see them as an inhibiting factor sometimes."*

## Discussion

In this study, we have identified barriers and facilitators for physical fitness training in orthopedic GR, from the perspective of patients, their relatives and professionals. We found that the barriers and facilitators were multi-factorial, with five main themes of program, patient, family, staff and organization factors. These themes are sometimes interrelated, and some barriers fitted in more than one theme. Insights into these factors can help to develop strategies to improve physical fitness training in orthopedic GR and tailor physical fitness training to a patient’s individual situation and needs.

Although research on barriers and facilitators has been conducted regarding the participation in self-care or self-management of older patients in the hospital [12,19], as well as participation in physical exercise in home-dwelling or residential older adults [10,11,13], no research has been conducted on barriers and facilitators of physical fitness training in orthopedic GR. In the abovementioned studies, the factors concerning the exercise program itself mainly concerned the content and form of the activity. For example, the studies of Gebhard and of Aro found group-based activities as being both motivating and hindering participation in physical activity. That can offer the opportunity to socialize, but it can also be confronting to see others performing better or worse [11,13]. The absence of factors related to goal setting, assessment and evaluation in the aforementioned studies can be explained by their settings, where the aim was not an improvement in functioning or participation as in GR.

All mentioned studies found patient factors as barriers and facilitators; for example, comorbidities [10,11,13], pain [10–12], good initial fitness [10,19] and self-efficacy [10,11]. In general, patient factors reported by other studies largely match those in our study, although we also found facilitators related to rehabilitation, such as adequate expectations and preparation for the rehabilitation process, as well as the patient taking on an active role and showing ownership.

Family factors are hardly mentioned in the aforementioned studies. Only Gebhard et al. found family support to be a motivating factor for the participation in physical activities among people with dementia in residential care settings [11]. A new topic found in our study is the relatives themselves practicing with the patient, as well as the factors influencing this.

All studies found staff factors such as motivating staff members who encourage physical activity [11–13,19] or – on the contrary – a physician who does not advise participating in it [10]. Only Chan et al. found heavy staff workload to be a barrier in their study on the participation of patients in self-care in a hospital setting, whereas the other studies did not report workload as a barrier [12]. A factor that we found that was not mentioned in other studies is staff knowledge and competence; for example, on how to assess the appropriate training level, and how to interpret pain in a patient.

Organization factors were also found in all studies. They concerned the layout of the house or the ward, the availability of equipment and the organization of care. For people with dementia, the feeling of being locked up is felt as a barrier for physical activity [11]. Hospitalized patients were hindered in their activities by the feeling of being a guest who is dependent on hospital procedures and not expected to take initiative in physical activities [12,19]. An interesting facilitator that the study of Schutzer et al. [10] reported – but was not found in our study – is the use of prompts such as telephone calls and emails as a nudge to participate in a program. The use of prompts – adapted to the GR setting – could be considered to enhance the performance of physical fitness training. Novel factors in our study are the topics related to multidisciplinary collaboration and communication between various stakeholders, such as family and GR professionals. Good collaboration and communication can contribute to the alignment of activities, resulting in a coherent training program in which all stakeholders play their role in the execution of physical fitness training.

Although our results are generally consistent with findings of studies in other settings, we also found barriers and facilitators that are unique for the setting of training physical fitness in GR. Patients recovering from a sudden decline in functioning have a targeted goal of improvement and wish to return home, which involves a multidisciplinary team and treatment program. Another difference of our study with the existing literature is that the other studies solely used the patients’ perspective, whereas we added the perspectives of their relatives and professionals. These differences explain – for example – the different program factors (like setting goals and factors for assessment and evaluation, as well as practicing outside of therapy) and factors concerning the organization of care (with a strong role of multidisciplinary collaboration), as well as the family factors that we found, which were absent in most other studies.

This exploration found no factors related to implementation. This is in accordance with our expectations because we investigated the current practice and not the implementation (strategy) of a specific intervention.

It was remarkable that one of the interviews lasted only 14 min, much shorter than the other interviews which lasted roughly between 30 and 60 min. This interview was conducted with a relative who had little time to visit the patient and had little knowledge of the patient’s rehabilitation trajectory.

### Strengths and limitations of the study

A first strength of our study is the fact that we used the contribution of patients, their relatives and GR professionals, thus providing a broad perspective on barriers and facilitators for physical fitness training and the process of GR. A second strength of our study is the triangulation of methods, which – combined with the aforementioned triangulation of viewpoints – enabled developing the most diverse and rich dataset possible in a limited timeframe. The interviews were held in triads around a patient (patient, informal caregiver and responsible nurse) and allowed exploring barriers and facilitators in an individual rehabilitation process in greater depth. The focus groups with GR professionals have the advantage of offering the broader scope of professionals who have experienced multiple rehabilitation trajectories. Moreover, the interaction between various participants enables complementing each other and prompting discussion. A third strength is the fact that the interviews took place during the inpatient rehabilitation trajectory of the participating patients, which minimized recall bias. Finally, the use of an implementation model covering the evaluation of barriers and facilitators appeared to be very helpful to develop an interview guide and build the preliminary framework for our results.

Limitations of our study include the fact that we did not recruit participants until saturation, and that we did not reach the desired number of interviews and focus groups. Hence, this may not provide a complete picture of the relevant factors. The included patients are probably not representative of the study population, being all female, living alone and their informal caregivers being their children. Initially, we intended to perform targeted sampling, for example to include both men and women, or to include patients with different kinds of (co) morbidity. As the inclusion appeared to be very difficult, we were happy with all included patients. Moreover, we did not systematically survey comorbidities and probably patients with cognitive problems are under-represented. This might limit the generalizability of the results as it comes to the patient perspective. Another limitation of our study is that for patients and their relatives the isolated focus on physical fitness training seemed difficult, as it is an integrated part of the overall rehabilitation process. In the focus groups, physical fitness training (or muscle strength and endurance training) was a known concept. For this reason, we chose to code all quotes that referred to activities that could influence physical fitness as ‘physical fitness training’, such as all quotes about walking, climbing stairs and cycling. However, it is possible that some of these quotes were not strictly related to the training of physical fitness. A last potential limitation is that both the interviewer and the moderator are elderly care physicians with professional experience in GR, which could have influenced the findings of the (focus group) interviews. However, as physicians, neither of them was involved in physical fitness training in GR and their experience with the setting was also an advantage. The two main coders were never involved in GR.

The resulting barriers and facilitators of this study reflect experiences and opinions of the participants. Further research should focus on weighing the found barriers and facilitators for their impact on the execution of physical fitness training quantitatively in a larger group of participants, developing a feasible guidance for daily practice, and testing the effect of this guidance in terms of adherence to the existing physical fitness training guidelines.

## Conclusions

To conclude, in this study we have offered insights into the barriers and facilitators of physical fitness training from the perspective of patients, their relatives and GR professionals. This overview of barriers and facilitators enables multidisciplinary teams to develop strategies to improve physical fitness training in two ways. First, the knowledge about barriers and facilitators at the level of the program, staff and organization can be used to design improvements within their organization and their interventions; for example, by implementing person-centered goal setting, taking care of well-trained staff, critically evaluating the training volume within the rehabilitation program, and improving the communication with patients and family about the GR process. Second, individual training programs can be better tailored to a patient’s circumstances and needs, taking into account the barriers and facilitators found at the patient and family level. Future research should focus on the development and testing of a feasible guidance for daily practice.

## Data Availability

The participants of this study did not give written consent for their data to be shared publicly, so due to the sensitive nature of the research supporting data is not available.
